# Self-Myofascial Vibro-Shearing: a Randomized Controlled Trial of Biomechanical and Related Changes in Male Breakdancers

**DOI:** 10.1186/s40798-018-0128-1

**Published:** 2018-03-27

**Authors:** Christopher-Marc Gordon, Sophie Manuela Lindner, Niels Birbaumer, Pedro Montoya, Rachel L. Ankney, Frank Andrasik

**Affiliations:** 1 CIT Research Institute, Ahorn Str. 31, 70597 Stuttgart, Germany; 2 Institute of Medical Psychology and Behavioral Neurobiology, University of Tübingen, Tübingen, Germany; 3 Research Institute on Health Sciences (IUNICS), University of Balearic Islands, Palma, Spain; 4 Department of Psychology, University of Memphis, Memphis, TN USA

**Keywords:** Tool-assisted self-myofascial release, Fascia-ReleaZer®, Tissue stiffness, Tissue elasticity, Range of motion, Pain pressure threshold, Breakdancer, IASTM

## Abstract

**Background:**

This randomized controlled trial explored the practicality and effectiveness of a novel tool-assisted self-help device, one that combines vibrational oscillation, leverage, and the shearing effect from the edges, for promoting meaningful changes in key biomechanical tissue indices and related parameters.

**Methods:**

One hundred and thirteen male breakdancers were randomized to an intervention or control group. Individuals assigned to the intervention group performed the self-help treatment on the quadriceps and the iliotibial band of their right thighs for 8 min, while individuals assigned to the control condition merely sat quietly during this period. Various primary outcome measures (e.g., elasticity, stiffness, range of motion, pain pressure threshold sensitization, and blood flow) were assessed before and after the intervention for each participant, with position and posture being standardized throughout. Subjective sensations and a measure selected to assess for potential experimental demand effects, serving as secondary measures, were also administered pre- to post-treatment.

**Results:**

Stiffness was significantly reduced for both structures (*p* < 0.001), elasticity and flexibility of the quadriceps were increased significantly (*p* < 0.001 for each), sensitization was significantly lessened (*p* < 0.001), and local temperatures increased to a significant degree as well (*p* < 0.001) when comparing change scores following application of the self-help tool on the treated thighs to those on the untreated thighs. Participants using the self-help tool reported their treated leg as being more relaxed, light, and stable.

**Conclusions:**

The vibro-shearing manipulation with a muscle-fascia tool resulted in significant improvements in various objective mechanical tissue properties, range of motion, and pain desensitization in healthy, well-conditioned dancers. These promising effects for a new tool-assisted self-treatment indicate further basic investigations are warranted, as are pilot investigations with patient populations.

**Electronic supplementary material:**

The online version of this article (10.1186/s40798-018-0128-1) contains supplementary material, which is available to authorized users.

## Key Points

- Vibro-shearing self-manipulation with the Fascia-ReleaZer® is a technique that reduces myofascial stiffness and pain sensitization.
- The self-manipulation tool increases microcirculation, elasticity, and range of movement.
- The subjective parameters of lightness and relaxation increased significantly through the treatment.

## Background

Self-myofascial release (SMR) is becoming increasingly popular both in the amateur and professional sports field. SMR is a type of myofascial release that is performed by individuals themselves, most often using a foam roller [1–3] or a roller massager [4–6]. SMR appears to have a wide range of effects, most typically increasing flexibility acutely [4–6] and chronically [7, 8] with respect to changes in joint range of motion (ROM), although it has also been utilized to reduce delayed onset muscle soreness (DOMS) [2, 6, 9], affect arterial and vascular endothelial function [3], and modulate autonomic nervous system activity [10].

More recently, therapeutic myofascial and soft tissue manipulation tools have incorporated devices that have firmer edges to increase leverage and/or vibration features to enhance effects. An evidence-based form of instrument-assisted soft tissue mobilization, called the Graston® Technique (GT), utilizes a stainless-steel tool to localize and treat soft tissue restrictions. Developers of the GT recommend that the edges of the instrument be prepared at a 30° to 60° angle to more effectively and efficiently address soft tissue lesions and fascial restrictions. Studies utilizing the GT have reported producing a localized inflammatory response, reducing scar tissue, and breaking down existing scar tissue in people with soft tissue restrictions [11, 12].

Vibration features have been used extensively since the beginning of the twentieth century in instrument-assisted techniques to mechanically stimulate myofascial tissue [13, 14]. Low-intensity vibratory massage (VM), with a 15–50-Hz frequency band, has been shown to increase oxygen uptake, blood and muscle oxidation, local and general blood circulation, local temperature in massaged tissues, and muscle enzyme activation. Other effects include marked general relaxation, relaxation of myofascial tissues, decrease of emotional tension, and a general sedative effect [15].

To date, no studies have investigated the value of adding a vibration component to the shearing effect of SMR tools. Shearing is defined as a mechanical force that acts on an area of skin in a direction parallel to the body’s surface. Shearing is affected by the amount of pressure exerted, the coefficient of friction between the materials contacting each other, and the extent to which the body makes contact with the support surface [16]. This study was designed to assess the clinical utility of a new self-help tool for treating myofascial tissue that combines a number of the aforementioned features—vibrational oscillation, leverage, and the vibro-shearing effect—in a novel self-help instrument called the Fascia-ReleaZer® (FR). This tool has the potential added advantage of having the user apply the desired pressure in the absence of external support, unlike foam rolling and related approaches that require the device to be pressed against a firm object.

The specific aim of this study was to explore the practicality and preliminary effectiveness of this new self-help tool by examining biomechanical tissue changes and other associated effects when this device is used. We selected a sample of individuals, experienced breakdancers, known to be particularly well fit. This was done to minimize potential biasing effects and thus provide a strong test of the value of FR. In this investigation, we selected the quadriceps muscle (QM) and the iliotibial band (ITB) as our test targets (see below why these targets were specifically selected).

Breakdancers, referred to as B-Boys within this community, were further specifically selected for participation in this exploratory investigation because the movement patterns in this dance style involve extremely strenuous physical activities, such as splits, spins, handstands, and tumbling, all varying in velocity, quality, and planes using all parts of the body fascia. Kicking of the legs and recruiting the myofascial tissues from the QM, as well as the ITB and the tensor fascia latae, produces the high velocity for the acrobatic maneuvers in breakdancing. All dance movements, such as jumping, spinning on the floor, or lifting, demand the perfect timing of the impulse, momentum, and rebound: the optimal timing in releasing the preloaded fascia [17, 18]. For an energetic and elastic movement, which is required in breakdancing, a dynamic catapult effect of the fascial fibers is necessary. Therefore, the elasticity of the fascial tissue is the key to its high capacity to store kinetic energy [19].

By addressing the inner body perspective, dance (breakdancing in particular) stimulates both the proprioception and interoception of the body. The highest density of proprioceptive and interoceptive receptors are found within the fascial tissue, because the fascial net plays an important role in the perception of inner sensations [20].

## Methods

### Overview of Study

Male breakdancers were randomly assigned to one of two experimental conditions: no-treatment control or a brief self-applied intervention using a specially designed myofascial manipulation tool. Various primary measures were taken from the right thighs of all dancers, with the intervention being applied to the right thigh only to the dancers assigned to the intervention group. A variety of primary outcome measures, described below, were collected from all dancers before and after intervention. Two final secondary measures were collected at pre- and post-treatment.

### Participants

One hundred thirteen (*N* = 113) male breakdancers were recruited and randomized into an intervention (*n* = 56) and a control group (*n* = 55). All participants fulfilled the following criteria: (1) healthy condition, (2) no physical preloading or physical execution, (3) skinfold measures of body fat at the ITB with a maximum of 10 mm, (4) dancing breakdance for a minimum of 1 year, and (5) age over 18 years. Exclusion criteria consisted of the presence of acute or chronic injuries concerning the legs and the hip area, pain in the treatment area, and acute inflammation and degenerative neurological illnesses or scars in the measuring area. Inspection of Table 1 reveals the groups were comparable with respect to all measures used for study inclusion. Written informed consent was obtained for all procedures, with participants being free to withdraw at any time without penalty. Of the total who consented to participate, two withdrew (one from each condition), reporting they became dizzy while sitting for an extended period. The study was conducted in a manner consistent with the Declaration of Helsinki and was granted for approval by the Ethics Committee of the University of Tübingen.

**Table 1:** Demographic information for the control and intervention groups

	Control (*N* = 55)		Intervention (*N* = 56)	
Variable name	*M*	*SD*	*M*	*SD*
Height (cm)	175.95	6.90	177.27	7.69
Weight (kg)	70.06	8.03	72.27	10.39
Breakdance experience (years)	7.94	6.02	8.17	5.18
Training (days/week)	3.56	1.42	3.65	1.23
Age (years)	23.85	5.02	23.20	5.02
Skinfold test (mm)	3.51	1.27	3.43	1.17

### Procedure

All participants were assessed with a common set of measures both prior to and following the time during which participants either received a brief intervention or sat and rested quietly for the same amount of time (control condition).

### Intervention Condition

**Fig. 1:**
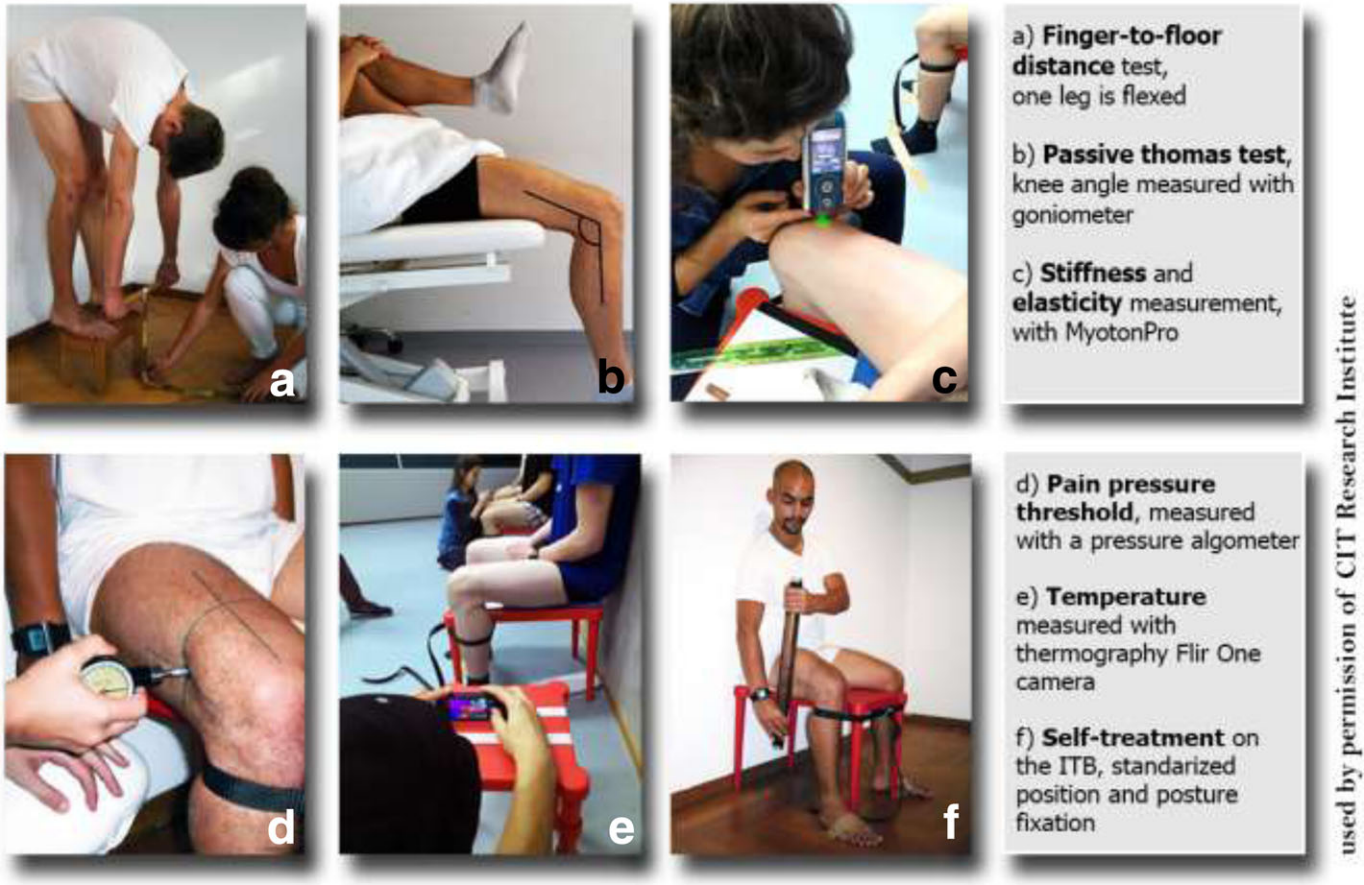
Protocol of assessments and self-treatment. **a** Finger-to-Floor Distance Test for the Hamstrings and Triceps Surae. **b** Passive Thomas Test for Hip flexors. **c** Objective biomechanical evaluation of tissues. **d** Pain Pressure Threshold (PPT) indicator of tissue sensitization. **e** Thermography, an indicator of micro-circulation. **f** Example of standardized self-treatment of the Iliotibial structures

Dancers assigned to receive the intervention performed an 8-min self-help treatment on their right thighs with the Fascia-ReleaZer®. The right thigh was first lightly lubricated with a vaseline-neutral oil. The opposite side was not oiled as this might have effected changes in the tissue. A standardized protocol was developed for the self-help treatment, which consisted of three short and three long linear strokes on the quadriceps muscle (QM) followed by three short and three long linear strokes on the iliotibial band (ITB). This procedure was repeated until 8 min had elapsed, gauging the time by a stopwatch placed on the wrist of the participant. The intervention tool was held and applied at a 45° angle to the treated tissue (see Fig. 1f). Patients were instructed to apply a pressure intensity that they subjectively perceived to be equivalent to a rating ranging from 3 to 5 on a numerical scale where 10 was the maximal value.

### Control Condition

Dancers assigned to the control condition were instructed to sit quietly in the standardized position and refrain from moving during the 8-min period when those in the intervention group applied the myofascial tool.

### Data Collection

#### Position and Marking of the Measuring Points

Position and posture were standardized for both groups, with participants being instructed to maintain the specified position and posture throughout. When necessary (which rarely occurred), they were corrected by the study examiners. Study position was controlled by having participants sit in a specific manner on a four-legged stool (58 cm length, 42 cm width, 43 cm height, UTTER IKEA). The back of the stool had contact with a flat wall. The lumbar spine of the participants had full contact with the wall, the pelvis was in a neutral position, and the participants were instructed to retain a straight posture. Both feet maintained full contact with the floor and were taped to the floor to help restrict any involuntary movement. The inside of the knees had full contact with the lateral sides of the stool. Both lower legs were attached with a strap to the front legs of the stool (see Fig. 1f).

A standardized protocol for marking the measuring points was performed by the same study examiner (the second author). A line was drawn from the epicondylus lateralis parallel to the femur on the strongest embossment of the ITB. The area revealing the highest level of stiffness was palpated and marked in the most anterior quarter of the ITB. A vertical line was drawn over the QM to this measuring point of the ITB. From the middle of the patellae, a line was drawn parallel to the rectus femoris muscle. The crossing of the two lines was the measuring point of the QM.

### Primary Outcome Measures

Objective measures were taken within multiple domains throughout the investigation in order to more fully explore and identify changes in response to our intervention, and these served as our primary measures. Assessors, all credentialed physical therapists, received 6 weeks of intensive training over the course of 14 pilot studies prior to conducting the assessments, with ongoing supervision by the first author provided throughout the investigation. All assessments were performed by the same study examiner (second author) and two further well-trained assessors, who met frequently during data collection to ensure continuity and prevent measurement drift.

### Biomechanical Tissue Changes

Key measures within this domain were collected with the MyotonPRO (Myoton AS; Estonia) [21, 22]. The measurement probe (*D* = 3 mm) was placed perpendicularly on the pre-marked skin areas above the muscle being measured, QM and the ITB (see Fig. 1c). The device was then lowered into the measurement position and held steady while it automatically performed the predefined measurement series (five single measurements with a recording interval of 1 s for each measuring point, using the average for data analysis). The method of myometry developed for measuring superficial skeletal muscles consists of (a) creating a constant pre-compression 0.18 N over the area of 7.1 mm^2^, followed by a brief (15 ms) mechanical impulse of 0.4 N to the contact surface of the skin; (b) recording the response of the tissue in a form of damped oscillation that is registered by an accelerometer in a form of an oscillation acceleration graph; and (c) subsequently conducting signal processing and computing for the variables of interest. Of the five available variables, we chose the two most pertinent to our study aims—elasticity and dynamic stiffness.

The logarithmic decrement of a tissue’s natural oscillation characterizes its elasticity, but more directly, it reflects the dissipation of the mechanical energy within an oscillation cycle, when tissues recover their shape after being deformed. Said another way, elasticity is the biomechanical property of a muscle that characterizes the ability to recover its initial shape after a contraction or removal of an external force of deformation. Elasticity is inversely proportional to the decrement. Therefore, as the decrement of a muscle decreases, the muscle elasticity increases. In theory, a decrement of 0 (zero) represents absolute elasticity. Elasticity is reported as the logarithmic decrement, while the inverse of elasticity is plasticity.

Dynamic stiffness [N/m] is the biomechanical property of a muscle that characterizes the resistance to a contraction or to an external force that deforms its initial shape. In the case of overly high stiffness, a greater effort is required from the agonist muscle to stretch a stiff antagonist, which leads to an inefficient economy of movement (see Additional file 1, relationship of the displacement oscillation and oscillation velocity in relation to the oscillation acceleration).

### Range of Motion

Three measures were collected with respect to this domain—the Modified Passive Thomas Test, Modified Thomas Test with Pressure—using a goniometer (KaWe Kirchner & Wilhelm, Germany), and the Modified Finger-Floor Distance Test—using a measuring tape.

#### Modified Passive Thomas Test (MPTT) and Modified Thomas Test with Pressure (MPrTT)

A standardized protocol was performed in order to obtain a value from the modified Thomas test. Study participants were asked to lie down on a table, with the left leg in knee flexion and then pulling this leg towards the chest. Participants were instructed to push the leg they were holding so far that the upper leg remained in full contact with the table. The angle of the knee was measured with a goniometer, providing a measure of the *MPTT* (see Fig. 1b). While in the same position, a measurement was taken with pressuring the lower leg into flexion until the first resistance was noticed, yielding scores for the *MPrTT*. All measurements taken with the goniometer were performed according to the technique described by Norkin and White [23].

#### Modified Finger-Floor Distance Test (MFFD)

A MFFD test was applied according to the procedures developed by Magnusson et al. [24]. Participants were asked to stand on a footstool (34 cm length, 19 cm weight, 23 cm height), grab a wooden stick with both hands while keeping both thumbs closed and both knees completely extended, and, from there on, flex the trunk towards the floor, with head and arms relaxed (see Fig. 1a). The participants were instructed to bend one leg after each other. Final flexion position was indicated by a sensation of muscular tension that caused initial hamstring discomfort. Finger-floor distance (in centimeter) was quantified using a measuring tape, yielding the MFFD value.

### Pain Pressure Threshold

The PainTest™ FPN 100 Algometer (Wagner Instruments, Greenwich, USA) is a device that measures deep pain pressure threshold (PPT) or tenderness resistance. More specifically, the algometer measures the pain pressure threshold when a particular site of the body is pressed with a rubber disk having an area of 1 cm^2^ [25, 26]. In the present study, the unit “kilogram” was used for quantifying PPT. The pressure algometer was placed over the marked measuring points of the QM only (see Fig. 1d). Study participants were extensively trained and instructed to verbally indicate the point at which point the pressure pain threshold that was steadily increased became “uncomfortable.” Higher pressure values thus indicate reduced pain pressure threshold sensitivity.

### Measurement of Tissue Temperature

Two distinct measures of temperature were collected over the ITB to serve as proxies for superficial blood flow—ITB thermography and ITB thermometer. As little is known about the preferred approach for assessing this aspect, we opted to collect both for comparability and to provide a measure of quality control.

#### ITB Thermography

The FLIR ONE™ thermography camera (FLIR Systems, Gmbh, Frankfurt, Germany) is used to take thermographic pictures and differentiates the temperature with a color scale. A normal daylight photograph, simultaneously taken with every thermographic picture, is used for documentation purposes. The device consists of an attachment for the iPhone 5 and is used together with the FLIR ONE™ App. Its measuring sector ranges from − 20 to 120 °C, with a sensitivity of ± 0.1 °C (the smallest absolute amount of change that can be reliably detected).

Changes in superficial temperature were recorded with this thermography camera, while aimed at the ITB measuring point. The position of the thermography camera was standardized by positioning the camera on a chair at a height of 48 cm (approximately at the position of the participant’s ITB) at a distance 60 cm away from the measuring chairs. Strips of tape were placed on the floor to mark the position of the chairs as well as the table for the thermography camera in order to standardize the distance. The sensor of the camera was directed towards the measuring point on the ITB in order to perform a spot measurement, and a calibration was performed before taking a picture (see Fig. 1e). The measuring values were recorded by the scientific assistant.

#### ITB Thermometer

A non-contact clinical thermometer (FT 90, Beurer, Ulm, Germany) was used to measure the superficial temperature for control of the thermography camera. Temperature values between 22 and 80 °C can be recorded with a sensitivity of ± 0.3 °C temperature units with this device. The thermometer was placed 2 to 3 cm in front of the measuring points at the ITB. The measuring values were recorded by the scientific assistant.

### Secondary Outcome Measures

Two secondary measures (Physical Sensations Questionnaire and Modified Profile of Mood States) were administered pre- to post-treatment in order to assess reactions to the brief treatment and preliminarily determine if demand (or reactivity) effects might have influenced the findings.

### Physical Sensations Questionnaire (PSQ)

This questionnaire was constructed by the investigators to inquire about the physical sensations experienced by participants pre- to post-intervention and was completed while standing on the right leg alone. All participants were asked to rate their subjective sensations of relaxation, lightness, strongness, and stability, on a scale that ranged from zero (“does not apply”) to ten (“does apply”) for each item. (No analyses were conducted to examine the psychometric properties of this investigator-created instrument.)

### Modified Profile of Mood States (POMS) Questionnaire

The modified *POMS* was administered to all participants pre- to post-trial to permit us to assess potential influences due to mere participation alone (e.g., demand or reactivity effects). We selected a modified German short version [27] of the original “Profile of Mood States” [28], a standardized psychological test formulated to assess momentary mood states. The 19 items contained within the version we used inquire about varied momentary feelings of respondents, with each being rated on a numeric scale, from 1 to 7, with the values described as “Not at All” (1), “Very Poor,” “Poor,” “A Little,” “Moderately,” “Quite a Lot,” or “Extremely” (7). Five subscales are computed for analysis—sadness, despair, tiredness, positive mood, and anger. This measure has acceptable psychometric support (Cronbach’s alpha ranges from *α* = .83 to .94; factorial, differential, and construct validity are reported as acceptable as well).

### Overview of Data Analysis

A series of *t* tests for independent groups was conducted for all 11 primary pre-treatment (baseline) values to assess equivalency of the two groups prior to intervention. This was followed by a multivariate analysis of variance (MANOVA) comparing the intervention (treated) and control groups with respect to all primary outcome measures (biomechanical tissue measures, range of motion, pain pressure threshold, and bloodflow) in one integrated analysis, using change scores (post-treatment minus pre-treatment values) as the unit for analysis in order to provide the most sensitive test of effects. A second MANOVA was conducted to address group differences with respect to the secondary outcome measures pertaining to perception of physical sensations and mood states, both collected prior to and at the end of the study (again using change score). MANOVA was chosen based on the recommendations of both Pallant [29] and Tabachnick and Fidell [30] that it is the most appropriate procedure to use when conducting analyses of variance with multiple dependent variables (DVs) as it can be used to detect which DVs are influenced by the manipulation. Multivariate analysis also protects against inflated type I error due to multiple tests, especially when DVs are correlated (which was the case here). The inclusion of covariates was judged unnecessary because only one primary outcome measure revealed a significant difference between groups (to be discussed later).

Prior to the MANOVA analyses, data were screened in SPSS 24 to ensure that the assumptions for this approach were met, following recommendations by Pallant [29] and Tabachnick and Fidell [30]. Univariate outliers were screened by variable, with outliers classified as those falling above or below 3 standard deviations of the mean. Multivariate outliers were screened separately for each planned analysis by conducting a linear regression with participant ID as the independent variable (IV) and all primary outcome dependent variables (DVs) for the planned analysis as the regression DVs. Outliers were classified as those with Mahalanobis’ distances falling outside of the chi-square threshold, consistent with the recommendations of Pallant [29] and Tabachnick and Fidell [30]. Both univariate and multivariate outliers were removed from analysis by applying these tests, and the resulting sample size is reported separately for each MANOVA analysis. As identified outliers varied as a function of each major analysis, the sample sizes reported per condition in the data table summaries vary somewhat.

## Results

Table 2 presents the findings when comparing pre-treatment values for all primary dependent measures for those serving as controls versus those receiving treatment. All but one test (that for the thermography measure) revealed no significant differences. A follow-up two-way ANOVA (period: pre- versus post-test × experimental condition: control versus intervention) for this measure revealed the difference at pre-treatment, although statistically significant, was substantially smaller in magnitude than the difference at post-treatment. Consequently, we opted not to employ any corrections for this, such as covariate analyses.

**Table 2:** Means, standard deviations (within parentheses), *t* test values, and significance levels comparing control versus intervention groups for all dependent measures at pre-treatment

Dependent variable	Control (*N* = 55)	Intervention (*N* = 50)	*t* test	Significance
	Mean (SD)	Mean (SD)		
Elasticity QM	.867 (.100)	.851 (.117)	.728	.468
Elasticity ITB	.836 (.095)	.820 (.104)	.831	.408
Stiffness QM	455.35 (74.50)	463.38 (103.77)	− .452	.653
Stiffness ITB	428.58 (69.98)	424.30 (51.95)	.358	.721
MPTT	60.18 (11.06)	58.86 (9.96)	.644	.521
MPrTT	104.55 (8.98)	104.28 (9.67)	.145	.885
MFFD	26.86 (10.39)	25.76 (9.40)	.571	.569
PPT QM	9.65 (4.29)	11.14 (4.33)	− 1.769	.080
PPT ITB	8.95 (4.01)	9.79 (3.97)	− 1.072	.286
Thermography ITB	29.18 (1.57)	29.93 (1.31)	− 2.682	.009
Thermometer ITB	31.70 (.94)	31.95 (.93)	− 1.343	.182

Results for both MANOVAs demonstrated significant multivariate differences based on Pillai’s trace, as reported in Table 3. Pillai’s trace is reported in lieu of Wilks’ lambda as recommended when assumptions, in this case a significant Box’s test, are violated [29]. Given significant findings were obtained for both multivariate tests, tests of between-participant effects were then investigated for each MANOVA to determine more precisely the sources of the significance.

**Table 3:** Multivariate analyses of change scores for primary and secondary measures

Test	Pillai’s trace	*Df*	*df* error	*F*	Partial *η*^2^
Primary measures	.788	11	83	28.10	.79
Right thigh					
Post-test minus pre-test					
PSQ and POMS	.323	9	89	4.72	.32
Post-test minus pre-test					

All *p* values < .001

The first MANOVA revealed a significant difference between groups for all of the primary outcome post-test minus pre-test difference scores, aside from elasticity in the ITB. Effect sizes based on partial eta squared, using Cohen’s [31] benchmarks of .01, .06, and .14 for small, medium, and large effects, respectively, indicated large effects for biomechanical tissue changes (with the exception of elasticity in the ITB), large effects for range of motion, medium to large effects for PPT, and large effects for blood flow (see Table 4, group differences in right thigh change from pre- to post-test by condition).

**Table 4:** Group differences in right thigh change from pre- to post-test by condition

Dependent variable	*df*	*df* error	*F*	Partial *η*^2^	Group	Means	95 % C.I.	
							Lower	Upper
Elasticity QM	1	93	29.18	.24	Control	− .006	− .022	.009
					Intervention	− .067	− .083	− .050
Elasticity ITB	1	93	.48	.01	Control	.005	− .009	.019
					Intervention	− .002	− .017	.013
Stiffness QM	1	93	48.96	.35	Control	− 2.90	− 12.57	6.77
					Intervention	− 52.96	− 63.36	− 42.55
Stiffness ITB	1	93	78.68	.46	Control	− 5.80	− 11.98	.38
					Intervention	− 46.36	− 53.02	− 39.71
MPTT	1	93	31.65	.25	Control	− .24	− 2.10	1.63
					Intervention	7.55	5.53	9.56
MPrTT	1	93	82.25	.47	Control	− 2.84	− 4.44	− 1.25
					Intervention	7.84	6.12	9.56
MFFD	1	93	67.26	.42	Control	.77	.10	1.43
					Intervention	− 3.37	− 3.99	− 2.56
PPT QM	1	93	13.63	.13	Control	− .06	− .75	.63
					Intervention	1.82	1.08	2.56
PPT ITB	1	93	16.99	.15	Control	− .59	− 1.35	.16
					Intervention	1.72	.90	2.53
Thermography ITB	1	93	103.96	.53	Control	− .18	− .52	.16
					Intervention	2.38	2.01	2.75
Thermometer ITB	1	93	81.38	.47	Control	− .51	− .88	− .14
					Intervention	1.98	1.58	2.39

Control *n* = 51, intervention *n* = 44; all *p* values < .001 for all measures, with the sole exception being elasticity ITB (*p* = .49)

The second and final MANOVA, again using change scores as the DV, revealed a significant difference between groups on PSQ relaxation and lightness, with partial *η*^2^ indicating large effect sizes for both measures. Results were not significant for either stability or strongness, although mean differences, along with a *p* value of .06, indicate a potential difference between groups on stability. Minimal differences were found for the POMS measures, with only POMS tiredness demonstrating a significant small to medium effect (see Table 5, group differences in secondary outcome measures from pre- to post-test comparing those assigned to the control condition versus the intervention condition).

**Table 5:** Group differences in secondary outcome measures from pre- to post-test by condition

Dependent variable	*df*	*df* error	*F*	Sig	Partial *η*^2^	Group	Means	95% C.I.	
								Lower	Upper
PSQ relaxation	1	97	20.20	< .001	.17	C	.447	− .321	1.215
						I	2.846	2.116	3.576
PSQ stability	1	97	3.74	.06	.04	C	− .064	− .755	.627
						I	.865	.208	1.522
PSQ strongness	1	97	1.81	.18	.02	C	− .532	− 1.184	.120
						I	.077	− .543	.696
PSQ lightness	1	97	35.72	< .001	.27	C	.277	− .448	1.001
						I	3.288	2.599	3.978
POMS sadness	1	97	.20	.65	.00	C	− .149	− .276	− .022
						I	− .109	− .230	.012
POMS despair	1	97	.19	.67	.00	C	− .099	− .217	.018
						I	− .064	− .176	.048
POMS tiredness	1	97	5.91	.02	.06	C	− .239	− .470	− .008
						I	− .630	− .850	− .410
POMS positive mood	1	97	2.14	.15	.02	C	− .113	− .329	.311
						I	.106	− .099	.029
POMS anger	1	97	.30	.58	.00	C	− .071	− .171	.029
						I	− .109	− .204	− .014

Control (C) *n* = 47, intervention (I) *n* = 52

## Discussion

The main aim of the present study was to determine whether meaningful changes would occur in objective mechanical tissue properties and related measures when young, healthy breakdancers applied a self-help treatment with a muscle fascia tool. After 8 min of use, elasticity increased in the quadriceps, stiffness (for the QM and the ITB) and pain sensitivity decreased, ROM improved for the QM and hamstrings, and local temperature values increased (reflecting improved blood flow). Although the intervention was very brief in duration, some differences were found with respect to perceived sensations, all of which favored those receiving the intervention. The absence of major differences on the POMS between those dancers assigned to the intervention condition and those assigned to the control condition suggests the observed findings are unlikely to have occurred due to participants’ awareness of them being observed or taking part in an experiment (i.e., demand or reactivity effects). Our findings are further strengthened by the fact that the demographic data of both groups were similar and comparable. The standard deviations for age, size, weight, training experience, training intensity, and training duration were small. Further, measurements of improvement were fairly consistent across all parameters for the treated legs only, with the control (untreated) legs basically remaining unchanged.

Stiffness reduced, while elasticity increased in the treated thigh. Elasticity of the ITB of the right thigh, however, did not change. The failure to find a change in the elasticity of the ITB is not surprising given that a certain level is required for lateral knee stabilization. The ITB serves to reinforce the fascia lata and divides the QM from the hamstring. Its inner side is in continuity with the lateral intermuscular septum, while on its posterior side, the majority of collagen fibers of the ITB are in continuity with the intramuscular septa [32]. The myofascial force transmission between gluteus maximus and lower leg muscles via the fascial lata shows the important role of the ITB in the movement patterns of the lower extremities.

An increased range of movement was achieved in the treated QM (MPTT, MPrTT). Bradbury-Squires et al. [4] also found increases, ranging from 10 to 16%, for knee-joint ROM with applying a roller massager for 20 and 60 s on the QM. Although the ischiocrural muscles (MFFD) were not treated by the participants, a significant change pre- to post-treatment in ROM was found. A coherence between the ITB and the ischicrurale muscles was also observed by Kwak et al. [33]. Hence, loading the ITB alters the kinematics and contact pattern of the tibiofemoral joint similarly to loading the hamstrings.

The intervention tool, the Fascia-ReleaZer®, with its combination of vibrational oscillation, leverage, and specific edges for a shearing manipulation of the myofascial tissue, thus seems useful. However, as all features were combined, it is not possible to determine which component contributed most to the effects observed.

### Possible Effects Due to Vibrational Oscillation

The physiological mechanism of vibrational oscillation that decreases stiffness and increases elasticity in myofascial tissues is uncertain. Several studies have pinpointed the mechanoreceptors, mainly the Pacinian corpuscles in connective tissues, ligaments, and joints, and the primary endings of the muscle spindles that are particularly sensitive to vibration [34]. The restorative effect of rhythmic low-frequency mechanical oscillations is frequently attributed to improvements in circulation, enhanced capillary permeability, and the transport of metabolites accumulated during previous work. Some have hypothesized that vibration-induced increased ROM could be due to reduced passive muscle stiffness through a decreased number of residual cross-bridges, some of them being broken by the mechanical vibratory stimulation [14, 35].

Some researchers have reported anesthetic effects due to the vibrational massage [36, 37]. Our findings of pain desensitization, manifested as decreased subjective pain threshold pre- to post-self-treatment on the treated leg, corroborate these findings in the literature.

The PSQ values describing the treated leg as more relaxed, light, and stable are consistent with the findings of other studies describing a general relaxation, relaxation of myofascial tissues, a decrease of emotional tension, and general sedative effect through application of a roller massager [15].

### Possible Effects Due to Leverage

Participants were able to apply the pressure using their own hands. An individual modification of the pressure could be moderated according to the perceived subjective pain pressure. Comparing the present self-help tool with other SMR tools, such as foam rolling, the entire body weight has to be applied and modification of weight is more difficult to regulate. Consequently, in order to hold one’s own body weight over the treatment duration in other SMR tool applications, such as foam rolling, proper shoulder and core muscle stabilization and sufficient strength in the upper extremities are required [1].

Instrument-assisted soft tissue mobilization (IASTM) is a popular treatment for myofascial restriction. IASTM uses specially designed instruments to provide a mobilizing effect to scar tissue and myofascial adhesions. Several IASTM tools and techniques are available, such as the Graston® technique (GT). In comparison to the self-help methodology of the Fascia-ReleaZer®, the GT needs to be applied by a trained clinician and, as yet, has not been adapted for self-application [38]. The GT treatment is thought to stimulate connective tissue remodeling through resorption of excessive fibrosis, along with inducing repair and regeneration of collagen secondary to fibroblast recruitment. This, in turn, results in the release and breakdown of scar tissue, adhesions, and fascial restrictions. The Fascia-ReleaZer® tool may operate in a similar manner, as it is also applied with pressure and incorporates specific edge features. As these tools differ in a number of key respects (i.e., material, technique, and treatment protocol), comparative analyses will need to be designed in a way that takes these differences into account.

### Possible Effects Due to The Specific Edges

The Fascia-ReleaZer® tool has four different edges in order to reach different parts of the myofascial tissue depending on the desired technique, fast versus slow. Clinicians may consider the fast shearing technique of the Fascia-ReleaZer® a form of gua sha, but the treatment rationale, goals, and application differ. Gua sha, therapeutic surface frictioning that intentionally raises transitory petechiae and ecchymosis, is a traditional East Asian healing technique. A smooth, rounded edge is press-stroked into the flesh enough to contact the fascial layer but not so firm that it causes pain or discomfort. Modern studies confirm a thermoregulatory function involving surface microcirculation, wherein increased skin blood flow in subpapillary tissue layers effectively conducts away heat [39, 40]. Our hypothesis assumes that increased blood circulation is stimulated in order to improve the metabolism and blood flow of the treated structures. The significant thermometer temperature increases as well as our thermography findings support this notion.

## Conclusions

The vibro-shearing manipulation with a muscle-fascia tool resulted in significant improvements in the objective mechanical tissue properties. Pain desensitization, range of movement, and thermography improved significantly as well. The fact that mood states remained relatively constant suggests that reactivity or demand effects were not in operation. Tool-assisted self-treatment with the Fascia ReleaZer® shows preliminary evidence of being an effective treatment modality, one that warrants further research, with a possible next step being application with a clinical population. Assuming effectiveness within a clinical setting, another further direction might involve a component or dismantling design to help pinpoint the relative contributions of the various features of the device. Investigations aimed at providing greater understanding of effects on the cellular level seem worthy of pursuit as well. Finally, although not feasible in this study, use of assessors blind to condition would add increased rigor in future studies.

## Additional file

Additional file 1: Relationship of the displacement oscillation (S) and oscillation velocity (V) in relation to the oscillation acceleration (a). After the single mechanical impulse is delivered and quick-released under constant precompression, the tissue being measured responds immediately in the form of a damped oscillation, causing the co-oscillation of: a) a tissue being measured, b) the pre-compressed subcutaneous tissue layers above the tissue (i.e., superficial skeletal muscle), c) the testing-end, d) measurement mechanism, and e) accelerometer attached to the measurement mechanism. Damped oscillation of a soft biological tissue is registered in the form of an acceleration graph (a). (DOCX 133 kb)

## Abbreviations

ANOVA: Analysis of variance
B-Boys: Breakdancers
DOMS: Delayed onset muscle soreness
DV: Dependent variable
FR: Fascia-ReleaZer®
GT: Gaston® Technique
IASTM: Instrument-assisted soft tissue mobilization
ITB: Iliotibial band
IV: Independent variable
MANOVA: Multivariate analysis of variance
MFFD: Modified Finger-Floor Distance Test
MPrTT: Modified Thomas Test with Pressure
MPTT: Modified Passive Thomas Test
POMS: Profile of Mood States
PPT: Pain pressure threshold
PSQ: Physical Sensations Questionnaire
QM: Quadriceps muscle
ROM: Range of motion
SMR: Self-myofascial release
VM: Vibratory massage

## References

[1] Healey KC, Hatfield DL, Blanpied P, Dorfman LR, Riebe D, Hatfield DL (2014). The effects of myofascial release with foam rolling on performance. J Strength Cond Res.

[2] MacDonald GZ, Button DC, Drinkwater EJ, Behm DG (2014). Foam rolling as a recovery tool after an intense bout of physical activity. Med Sci Sports Exerc.

[3] Okamoto T, Masuhara M, Ikuta K (2014). Acute effects of self-myofascial release using a foam roller on arterial function. J Strength Cond Res.

[4] Bradbury-Squires DJ, Noftall JC, Sullivan KM, Behm DG, Power KE, Button DC (2015). Roller-massager application to the quadriceps and knee-joint range of motion and neuromuscular efficiency during a lunge. J Athl Train.

[5] Halperin I, Aboodarda SJ, Button DC, Andersen LL, Behm DG (2014). Roller massager improves range of motion of plantar flexor muscles without subsequent decreases in force parameters. Int J Sports Phys Ther.

[6] Jay K, Sundstrup E, Sondergaard SD (2014). Specific and crossover effects of massage for muscle soreness: randomized controlled trial. Int J Sports Phys Ther.

[7] Ebrahim A, Elghany A (2013). The effect of foam roller exercise and nanoparticle in speeding of healing of sport injuries. J Am Sci.

[8] Mohr AR, Long BC, Goad CL (2014). Foam rolling and static stretching on passive hip flexion range of motion. J Sport Rehabil.

[9] Pearcey GE, Brandbury-Squires DJ, Kawamoto JE, Drinkwater EJ, Behm DG, Button DC (2015). Foam rolling for delayed-onset muscle soreness and recovery of dynamic performance measures. J Athl Train.

[10] Chan YC, Wang TJ, Chang CC (2015). Short-term effects of self-massage combined with home exercise on pain, daily activity, and autonomic function in patients with myofascial pain dysfunction syndrome. J Phys Ther Sci.

[11] Gehlsen GM, Ganion LR, Helfst R (1999). Fibroblast responses to variation in soft tissue mobilization pressure. Med Sci Sports Exerc.

[12] Laudner K, Compton BD, McLoda TA, Walters CM (2014). Acute effects of instrument assisted soft tissue mobilization for improving posterior shoulder range of motion in collegiate baseball players. Int J Sports Phys Ther.

[13] Edge J, Mundel T, Weir K, Cochrane DJ (2009). The effects of acute whole body vibration as a recovery modality following high-intensity interval training in well-trained, middle-aged runners. Eur J Appl Physiol.

[14] Fiodorov VL (1971). Vibratory massage.

[15] Issurin VB (2005). Vibrations and their application in sport: a review. J Sports Med Phys Fitness.

[16] Bergstrom N, Bennett MA, Carlson CE (1994). Treatment of pressure ulcers.

[17] Cho CH, Song KS, Min BW, Lee SM, Chang HW, Eum DS (2009). Musculoskeletal injuries in break-dancers. Injury.

[18] Kauther MD, Wedemeyer C, Wegner A, Kauther KM, von Knoch M (2009). Breakdance injuries and overuse syndromes in amateurs and professionals. Am J Sports Med.

[19] Sawicki GS, Lewis CL, Ferris DP (2009). It pays to have a spring in your step. Exerc Sport Sci Rev.

[20] Schleip R, Müller DG (2013). Training principles for fascial connective tissues: scientific foundation and suggested practical applications. J Bodyw Mov Ther.

[21] Aird L, Samuel D, Stokes M (2012). Quadriceps muscle tone, elasticity and stiffness in older males: reliability and symmetry using the MyotonPRO. Arch Gerontol Geriatr.

[22] Mullix J, Warner M, Stokes M (2012). Testing muscle tone and mechanical properties of rectus femoris and biceps femoris using a novel hand held MyotonPRO device: relative ratios and reliability. Working Papers in Health Sciences.

[23] Norkin CC, White DJ (2016). Measurement of joint motion; a guide to goniometry.

[24] Magnusson SP, Simonsen EB, Aagaard P, Boesen J, Johannsen F, Kjaer M (1997). Determinants of musculoskeletal flexibility: viscoelastic properties, cross-sectional area, EMG and stretch tolerance. Scand J Med Sci Sports.

[25] Kinser AM, Sands WA, Stone MH (2009). Reliability and validity of a pressure algometer. J Strength Cond Res.

[26] Park G, Kim CW, Park SB, Kim MJ, Jang SH (2011). Reliability and usefulness of the pressure pain threshold measurement in patients with myofascial pain. Annals of Rehabilitation Medicine.

[27] Dalbert C (1992). Subjektives Wohlbefinden junger Erwachsener: Theoretische und empirische Analysen der Struktur und Stabilität. (The subjective well-being of young adults: theoretical and empirical analysis of structure and stability.). Zeitschrift für Differentielle und Diagnostische Psychologie (Journal of Differential and Diagnostic Psychology).

[28] McNair D, Lorr M, Droppleman L (1971). Mannual for the profile of mood states (POMS).

[29] Pallant J. SPSS survival manual: a step by step guide to data analysis using IBM SPSS. Maidenhead, Berkshire, England; New York, NY, McGraw Hill; 2013. p. 293–300.

[30] Tabachnick BG, Fidell LS (2007). Using multivariate statistics.

[31] Cohen J. Statistical power analysis for the behavioural sciences. Hillside: Lawrence Earlbaum Associates. New York: Routledge Academic. 1988.

[32] Stecco A, Gilliar W, Hill R, Fullerton B, Stecco C (2013). The anatomical and functional relation between gluteus maximus and fascia lata. J Bodyw Mov Ther.

[33] Kwak SD, Ahmad CS, Gardner TR (2000). Hamstrings and iliotibial band forces affect knee kinematics and contact pattern. J Orthop Res.

[34] Lundeberg T, Nordemar R, Ottoson D (1984). Pain alleviation by vibratory stimulation. Pain.

[35] Wakim KG, Basmajian JV (1985). Physiological effects of massage. Manipulations, traction and massage.

[36] Lau WY, Nosaka K (2011). Effect of vibration treatment on symptoms associated with eccentric exercise-induced muscle damage. Am J Phys Med Rehabil.

[37] Pournot H, Tindel J, Testa R, Mathevon L, Lapole T (2016). The acute effect of local vibration as a recovery modality from exercise-induced increased muscle stiffness. J Sports Sci Med.

[38] Cheatham SW, Lee M, Cain M, Baker R (2016). The efficacy of instrument assisted soft tissue mobilization: a systematic review. J Can Chiropr Assoc.

[39] Braun M, Schwickert M, Nielsen A (2011). Effectiveness of traditional Chinese “gua sha” therapy in patients with chronic neck pain: a randomized controlled trial. Pain Med.

[40] Kwong KK, Kloetzer L, Wong KK (2009). Bioluminescence imaging of heme oxygenase-1 upregulation in the Gua Sha procedure. J Vis Exp.

