# Supplementary material for: Self-Myofascial Vibro-Shearing: a Randomized Controlled Trial of Biomechanical and Related Changes in Male Breakdancers
Source: Sports Med Open. 2018 Mar 27;4:13. doi: 10.1186/s40798-018-0128-1 (PMC5876229; doi:10.1186/s40798-018-0128-1)
Supplement: Supplementary file 1 — Relationship of the displacement oscillation (S) and oscillation velocity (V) in relation to the oscillation acceleration (a). After the single mechanical impulse is delivered and quick-released under constant precompression, the tissue being measured responds immediately in the form of a damped oscillation, causing the co-oscillation of: a) a tissue being measured, b) the pre-compressed subcutaneous tissue layers above the tissue (i.e., superficial skeletal muscle), c) the testing-end, d) measurement mechanism, and e) accelerometer attached to the measurement mechanism. Damped oscillation of a soft biological tissue is registered in the form of an acceleration graph (a). (DOCX 133 kb) [file 40798_2018_128_MOESM1_ESM.docx]

**Additional File 1**.


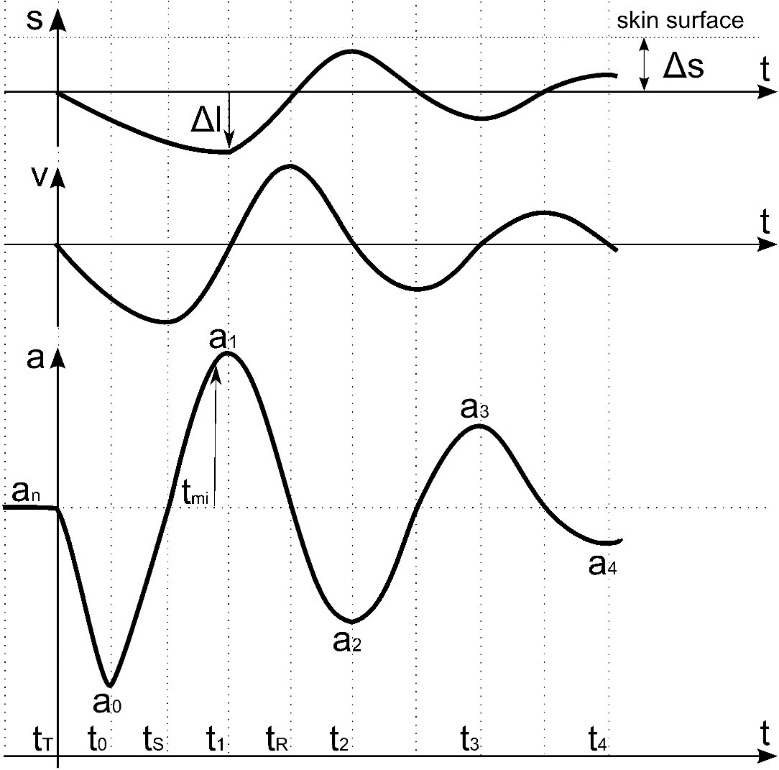


Additional File 1. Relationship of the displacement oscillation (S) and oscillation velocity (V) in relation to the oscillation acceleration (a). After the single mechanical impulse is delivered and quick released under constant precompression, the tissue being measured responds immediately in the form of a damped oscillation, causing the co-oscillation of: a) a tissue being measured, b) the pre-compressed subcutaneous tissue layers above the tissue (ie superficial skeletal muscle), c) the testing-end, d) measurement mechanism, e) accelerometer attached to the measurement mechanism. Damped oscillation of a soft biological tissue is registered in the form of an acceleration graph (a).

The legend of Additional File 1:

| Symbol | Description |
| --- | --- |
| a | Acceleration of oscillation [mG] |
| a0 | Maximum acceleration |
| a1 | Maximum displacement ie maximum tissue resistance |
| a2 | Opposite displacement due to residual inertia |
| a3 | Maximum displacement of the second period of damped oscillation |
| ΔS | Pre-compression of subcutaneous tissues |
| Δl | Maximum displacement |
| mprobe | Mass of the measurement mechanism |
| S | Tissue oscillation - displacement [mm] |
| t | Time [ms] |
| tmi | The end of mechanical impulse |
| t0 | The time point where max acceleration is reached |
| tT | Start of the mechanical impulse |
| tr | The time point of tissue recovery to its original shape |
| ts | Zero acceleration ie maximum velocity |
| tt – t0 | The time of acceleration to increase from zero to maximum |
| t0 – ts | The time of acceleration to decrease from maximum back to zero |
| ts – t1 | The time to decrease from maximum velocity to zero velocity of deformation (tissue resistance equals with force of deformation) |
| t1 – tr | The time of acceleration to increase from maximum displacement to its original shape |
| tr – t2 | The time of acceleration to decrease from recovered original shape to the opposite displacement |
| t1 | The time point of maximum displacement |
| t2 | The time point of maximum opposite displacement |
| t3 | The time point of maximum displacement of the second oscillation |
| t2 – t3 | The time between two consecutive displacements |
| V | Velocity of oscillation [m/s] |
